# Dislocation of the Hip Joint Successfully Reduced by Manipulation Five Months after the Accident

**Published:** 1864-09

**Authors:** J. Newton Brown

**Affiliations:** San Jose


### DISLOCATION OF THE HIP JOINT

### SUCCESSFULLY REDUCED BY MANIPULATION FIVE MONTHS AND A HALF AFTER THE ACCIDENT.

By J. NEWTON BROWN, M.D., San Jose.

The subject of this paper was suggested by a case which came under my care while acting as one of the attendant physicians to the Infirmary of Santa Clara County, and on account of the success which attended an operation usually considered impracticable in such cases, I have thought it worthy of being reported.

Horatio N. Grant, aged 56, sanguino-nervous temperament, vigorous constitution, and in good health, was admitted to the Infirmary for an injury of the hip, which he had received five months and fifteen days previously, by being knocked down and run over by a horse. Upon examination it was found that the right femur was dislocated into the thyroid foramen, the hip was flattened, trochanter major depressed, the limb everted, abducted, and one and a half inches longer than that of the sound side. He walked with a cane, but was unable to use the limb in any manner which would necessitate motion of the thigh, except in a lateral and semicircular direction. He had been examined shortly after receiving the injury, by a physician, who he said "pulled at the limb and gave him some liniment." He had afterwards remained in bed about three weeks, receiving no further attention, and finally, after much suffering in traveling from place to place on foot, came to the Infirmary in the condition above described. A careful examination proved that the bone was exceedingly immovable except in one direction—viz: slight abduction, with very limited rotation, and that not without giving great pain. Taking into account the excellent physical condition of the patient, I determined to attempt reduction by manipulation, believing that if I failed in this, I could at least increase the mobility of the limb.

The patient was placed under the influence of chloroform, and when fully anæsthetized it was found that although the bone admitted of slight motion, it seemed to resist any efforts at flexion or adduction; and fearing, from the extent and firmness of the adhesions, that any attempt at reduction might result in serious laceration, I had almost determined to desist from further interference, but having been so earnestly solicited by the patient to undertake any thing, however hazardous, which might afford any chance of relieving him, I determined if possible to break up the adhesions, hoping that at least greater usefulness of the limb would be acquired. Flexing the leg upon the thigh, and placing my breast against the knee, I gradually threw my weight upon the knee joint, using the femur as a lever, and had the satisfaction of feeling the limb gradually move towards the patient's body, the adhesions giving way with quite an audible snapping and tearing sound. The manipulations were continued about ten minutes, and the mobility of the thigh greatly increased, but as there were still powerful muscular contractions, which seemed to increase with every movement of the femur, I placed the limb again in a horizontal position, and discontinued the manipulations. When the patient came from under the influence of chloroform, morphiæ acetas, and antim. et potass. tart. were administered, and cold lotions constantly applied to the hip. I was surprised the next morning to find that little, if any, constitutional disturbance had been produced, and the patient expressed himself as feeling quite comfortable. I now felt sanguine of being able to reduce the dislocation, and only feared adventitious deposit in the acetabulum. On the day following, chloroform was again administered, and the manipulations commenced as before. The adhesions continued to give way, and in twenty minutes the mobility of the limb was as great as could be attained from the unnatural position of the head of the bone. I now flexed the leg upon the thigh, and the thigh upon the pelvis, very slowly and cautiously carrying the knee over to the sound side and then across the abdomen, at the same time using considerable force in order to keep it as near the body as possible, and at this stage of the process the luxation was converted to the dorsum of the ilium; the limb was shortened, the toes inverted and resting upon the instep of the other foot. On making a second attempt, when I had arrived at this stage of the process when the knee was nearly on a line with the injured side, I abducted it gently, turned the toes outwards and the heel inwards, carrying the *foot* across the sound limb, making at the same time gentle oscillations of the thigh when the head of the bone slipped into the acetabulum, the foot came down and the deformity was removed. The feet were confined together, the limb bandaged, a full anodyne given, and with subsequent treatment in the way of light diet, evaporating lotions, &c., in three weeks the patient walked out into the yard with a cane, and in nine weeks from his admission to the Infirmary he was discharged cured. I have seen him since, nearly a year subsequent to the operation, and he walks as well apparently as any one: in short, is perfectly well. This case is interesting on account of the time which had elapsed previous to the operation, and as showing the practicability and superiority of "Reid's Method," as compared with the pulleys.

Dr. Reid's directions are as follows:

"Let the operator stand or kneel on the injured side, seize the ankle with one hand, the knee with the other, then flex the leg on the thigh, next strongly adduct it, carrying it over the sound one, and at the same time upward over the pelvis, by a kind of semicircular sweep, as high as the umbilicus; *then abduct the knee gently*, turn the toes outwards, the heel inwards, *and carrying the foot across the opposite and sound limb making gentle oscillations of the thigh*, when the head of the bone will slip into its socket."

Reid's method, in common with all improvements in surgery, has had its opponents, yet among those who have really investigated it, there are few who condemn, and many who award the praise which its merits deserve. Hamilton collected sixty-four cases in which it had been successful, and in sixteen of these cases manipulation succeeded after extension had failed. I have seen it resorted to in seven cases, varying in duration of time after the injury—from a few hours to 5½ months—and in every instance with favorable results. Three of these cases were on the dorsum of the ilium, two on the pubes, one in the ischiatic notch and one in the obturator foramen. One of these patients was 63 years old, the youngest about 36.

We do not wish to be understood as claiming infallibility for this method of reduction, for we will sometimes fail in reducing a dislocation of the hip, in spite of all our endeavors, either by traction or manipulation; but we do claim that all reducible cases can be successfully treated by Reid's method. The question comes in here—as to when, and under what circumstances, we should attempt reduction at all—this is a matter for the surgeon to decide.—*Pacific Medical and Surgical Journal.*

